# Sloping land use affects the complexity of soil moisture and temperature changes in the loess hilly region of China

**DOI:** 10.1371/journal.pone.0262445

**Published:** 2022-01-14

**Authors:** Chao Zhang, Min Tang, Xiaodong Gao, Qiang Ling, Pute Wu

**Affiliations:** 1 College of Hydraulic Science and Engineering, Yangzhou University, Yangzhou, Jiangsu, China; 2 Institute of Water-Saving Agriculture in Arid Areas of China, Northwest Agriculture and Forestry University, Yangling, Shaanxi, China; 3 College of Water Resources and Environmental Engineering, Zhejiang University of Water Resources and Electric Power, Hangzhou, Zhejiang China; Jinan University, CHINA

## Abstract

Various land use types have been implemented by the government in the loess hilly region of China to facilitate sustainable land use. Understanding the variability in soil moisture and temperature under various sloping land use types can aid the ecological restoration and sustainable utilization of sloping land resources. The objective of this study was to use approximate entropy (ApEn) to reveal the variations in soil moisture and temperature under different land use types, because ApEn only requires a short data series to obtain robust estimates, with a strong anti-interference ability. An experiment was conducted with four typical land use scenarios (i.e., soybean sloping field, maize terraced field, jujube orchard, and grassland) over two consecutive plant growing seasons (2014 and 2015), and the time series of soil moisture and temperature within different soil depth layers of each land use type were measured in both seasons. The results showed that the changing amplitude, degree of variation, and active layer of soil moisture in the 0–160 cm soil depth layer, as well as the changing amplitude and degree of variation of soil temperature in the 0–100 cm soil layer increased in the jujube orchard over the two growing seasons. The changing amplitude, degree of variation, and active layer of soil moisture all decreased in the maize terraced field, as did the changing amplitude and degree of variation of soil temperature. The ApEn of the soil moisture series was the lowest in the 0–160 cm soil layer in the maize terraced field, and the ApEn of the soil temperature series was the highest in the 0–100 cm layer in the jujube orchard in the two growing seasons. Finally, the jujube orchard soil moisture and temperature change process were more variable, whereas the changes in the maize terraced field were more stable, with a stable soil moisture and temperature. This work highlights the usefulness of ApEn for revealing soil moisture and temperature changes and to guide the management and development of sloping fields.

## Introduction

The loess hilly region is one of the most critical soil erosion areas in China, which constrains the ecological and sustainable development of agriculture [1, 2]. Ecological vegetation restoration is an effective method for controlling soil erosion, a strenuous task that needs to be executed over long time periods in the loess hilly region [3, 4]. Soil water and heat are important factors that influence vegetation development, characterized using soil moisture and temperature [5, 6]. Therefore, investigating the variability in soil moisture and temperature is important for vegetation restoration in the loess hilly region.

Many studies have demonstrated that land use is a key factor that affects the variations in soil moisture and temperature [7, 8]. Yu et al. investigated the seasonal variations in deep soil moisture under various land use types on the semi-arid Loess Plateau of China and reported that land use types had a significant impact on the spatial distribution of deep soil moisture at the hillslope scale [9]. Baboshkina et al. analyzed the moisture regime of chernozems in the Uymon intermountain depression landscapes with various agricultural use types, revealing the variability in soil moisture for different land use types [10]. Savva et al. evaluated the effects of both land use and vegetation cover on soil temperature in an urban ecosystem and suggested a significant difference in monthly average soil temperature between grasslands and forests [11]. Yan et al. investigated the microclimatic effects of various land use types in demonstration areas to combat karst rocky desertification and confirmed that land use types had a determinant effect on soil temperature [12]. However, most of these studies used traditional statistical analysis methods, such as variance, extreme value ratio, and coefficient of variation. The reliability of the evaluation results of the above-mentioned traditional statistical methods is significantly affected by the sample size, that is, the length of the data series. The larger the sample size, the more precise the evaluation result [13]. However, data series that can be obtained from field experiments are typically limited.

The change process of soil moisture and temperature presents a series of complexity due to the coupling of weather, vegetation coverage, and topography. Approximate entropy (ApEn), a nonlinear kinetic parameter, measures the regularity and unpredictability of time-series fluctuations, and indicates the possibility of new information in the series [14]. ApEn increases as the time series becomes more complex. In recent decades, ApEn has been developed and widely used for mechanical fault diagnosis, power system fault signal analysis, biomedicine, and electroencephalograms, owing to its adaptability, robustness, and strong anti-noise capability [15–17]. In recent years, ApEn has also been applied for the analysis of the regularity and complexity of soil moisture movements and temperature variations under various conditions. Zhou et al. estimated the soil moisture distribution in the 10 m deep vadose zone of the Badain Jaran Desert based on the entropy method and pointed out its usefulness in desert regions with extreme climatic conditions [18]. Wang investigated the complex characteristics of soil moisture and soil temperature at different depths in a snow‒soil composite system using the ApEn theory, which revealed that the complexity of the soil moisture and soil temperature series was consistent with the actual situation [19]. However, in the loess hilly region, the variable underlying surface due to the combined effects of precipitation, evaporation, runoff, and various land use types has resulted in complex spatial changes in soil moisture and temperature, and this variability is sensitive to the soil, atmosphere, vegetation, topography, etc.

In this study, four planting structures were implemented, including soybean sloping field, maize terraced field, jujube orchard, and grassland. The ApEn and two typical indicators of extreme value ratio and coefficient of variation were compared. The objectives of this study were to (1) investigate the variability in soil moisture and temperature under four land use types, and (2) evaluate the performance of different methods to investigate the characteristics of time-series soil moisture and temperature changes during two growing seasons in a normal precipitation year and dry year.

## Materials and methods

### Study area

The study area is located in the Yuanzegou watershed (37°13′38″–37°14′22″ N, 110°21′10″–110°21′32″ E) in the loess hilly region of northern Shaanxi, China. The region is characterized as a typical warm temperate continental monsoon climate zone, with a multi-year average precipitation of 497 mm and a multi-year average air temperature of 9.6°C. This region experiences severe soil erosion and ravines crisscross the landscape. The soil in the study area is loessal, with a loose texture, low erosion resistance, and low fertility, with a field capacity and wilting moisture of 0.25 and 0.07 cm^3^∙cm^-3^, respectively.

### Experimental design

Since the implementation of policies for returning farmland to woodland and grassland in the loess hilly region, a diverse range of land use and vegetation types has developed in the region, including a large area of cultivated land for planting maize, potatoes, and soybeans, grasslands formed by returned farmland, and scattered woodlands (jujube orchard, Caragana woodland, Robinia woodland, etc.). Thus, four planting structures of the soybean sloping field, maize terraced field, jujube orchard, and grassland were designed for two growing seasons, 2014 and 2015, at similar slope aspects (shady slope) and gradients (approximately 18°). Only one representative sloping field was chosen to characterize a land use type because there were few sloping fields with the same characteristics (vegetation, aspect, gradient, etc.) in the study area. The seeding densities of soybean and maize were 19.5×10^4^ and 9×10^4^ plants∙ha^-1^, respectively, and both were sown in late April and harvested in early October. Jujube trees were planted in 2003 with plant spacing of 2 m and row spacing of 3 m; they were in a full bearing period during the growing season. The grassland had been naturally restored from sloping farmland over 30 years ago. *Artemisia gmelinii* was the primary plant, and the associated plants were *Lespedeza davurica* and *Bothriochloa ischaemum*. The soil properties of the four experimental sloping land use types are listed in Table 1.

**Table 1:** Soil properties of experimental sloping land use types.

Sloping land use types	Depth (cm)	BD (g·cm^-3^)	Soil texture			K_sat_ (cm·d^-1^)
			Sand (%)	Silt (%)	Clay (%)	
Soybean sloping field	0–20	1.17 ± 0.15	21.0 ± 5.6	63.0 ± 3.7	16.0 ± 3.9	74.2 ± 20.6
	20–40	1.29 ± 0.11	19.5 ± 5.4	63.4 ± 2.4	17.0 ± 4.5	
	40–60	–	19.9 ± 3.9	65.0 ± 1.9	15.1 ± 3.2	
Maize terraced field	0–20	1.26 ± 0.11	17.7 ± 1.9	63.8 ± 2.3	18.5 ± 2.9	55.6 ± 10.4
	20–40	1.36 ± 0.07	16.6 ± 3.9	64.9 ± 1.7	18.6 ± 4.1	
	40–60	–	16.2 ± 4.1	63.3 ± 1.1	20.5 ± 4.2	
Jujube orchard	0–20	1.31 ± 0.12	23.7 ± 4.0	62.6 ± 1.9	13.7 ± 2.4	36.6 ± 9.6
	20–40	1.41 ± 0.10	21.6 ± 3.4	64.0 ± 1.9	14.4 ± 2.7	
	40–60	–	20.7 ± 2.2	64.2 ± 1.2	15.1 ± 2.5	
Grassland	0–20	1.28 ± 0.09	17.3 ± 2.8	63.6 ± 1.3	19.1 ± 3.4	35.3 ± 8.3
	20–40	1.28 ± 0.04	15.1 ± 1.7	62.9 ± 1.6	22.0 ± 0.7	
	40–60	–	14.9 ± 3.7	63.3 ± 1.3	21.8 ± 3.8	

Notes: BD: bulk density; Soil texture: Sand% (2–0.02 mm), Silt% (0.02–0.002 mm), and Clay% (<0.002 mm); K_sat_: saturated hydraulic conductivity. The data are the means ± SD of three replicate samples. Sample date: September 5, 2014.

### Soil moisture and temperature measurements

Two sets of automatic soil moisture and temperature monitoring devices were installed in the middle slope of each experimental plot along the same contour line at a distance of 10 m. The observation points in the soybean sloping field and maize terraced field were placed between crop rows, and the monitoring probes in the jujube orchard were buried 30 cm away from the jujube tree trunks. The EC-5 soil moisture sensor (Decagon Devices, Pullman, WA, USA) and the RR-7110 soil temperature sensor (Rainroot Scientific Ltd., Peking, CN) were used to measure soil volumetric moisture and temperature, respectively. The soil moisture sensor probes were buried at depths of 10, 20, 60, 100, and 160 cm. The probes of the soil temperature sensor were buried at depths of 10, 20, 40, 60, and 100 cm. Soil moisture and temperature were measured every 2 min during the vegetation growing season (May to October), and the data were recorded every 10 min. The soil moisture and temperature at the same depth at the two monitoring points in a certain land use type was averaged to characterize the soil moisture and temperature at that depth.

### Meteorological data

**Fig 1:**
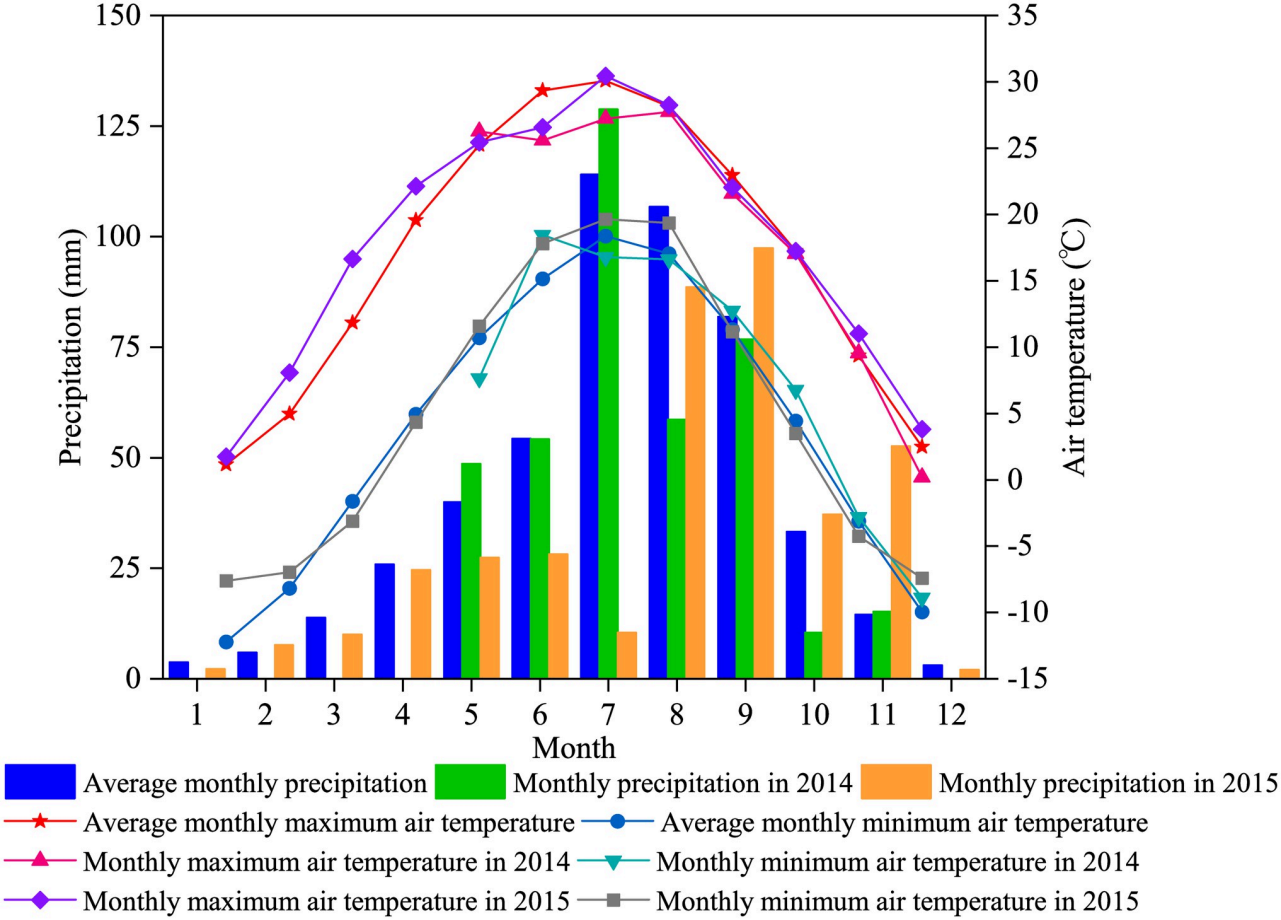
Monthly precipitation, and maximum and minimum air temperature in the study area for 2014 and 2015.

Meteorological data, including air temperature, air humidity, atmospheric pressure, solar radiation, wind speed, and precipitation in the study area were continuously measured using an automatic weather station (AR5 Automatic Weather Station, Avolon Scientific, Inc., Jersey City, NJ, USA). The monthly precipitation and air temperature (maximum and minimum) in 2014 and 2015 are shown in Fig 1. The total precipitation in the growing season (May to October) of 2014 and 2015 was 377.4 and 289.2 mm, representing a normal precipitation and a dry year, respectively, according to the method proposed by Hao et al. [20].

### Data processing

Previous studies have found that the soil moisture and temperature of the 0–20 cm soil layer under the four experimental sloping land use types are highly susceptible to the influence of precipitation and air temperature and fluctuate widely, whereas the change in soil moisture and temperature in the 20–60 cm soil layer is weaker than in the 0–20 cm soil layer, and there is an obvious lag in the response to precipitation and air temperature, and soil moisture and temperature below 60 cm change smoothly [21–23]. To quantitatively describe the vertical distribution characteristics of soil moisture and temperature in the four experimental plots, the soil moisture profile was divided into the surface layer (0–20 cm), middle layer (20–60 cm), and deep layer (60–160 cm); the soil temperature profile was also divided into the surface layer (0–20 cm), middle layer (20–60 cm), and deep layer (60–100 cm). Averaging the soil moisture and soil temperature at depths of 10 and 20 cm yielded the soil moisture and temperature of the surface layer. The soil moisture at 60 cm was considered as the middle layer’s soil moisture. The soil moisture in the deep layer was calculated by averaging the soil moisture at 100 and 160 cm. The soil temperature in the middle layer was calculated by averaging the soil temperatures at depths of 40 and 60 cm, and the soil temperature at 100 cm was considered as the soil temperature of the deep layer.

#### Descriptive statistics

The extreme value ratio (K_a_) and coefficient of variation (C_v_) were used to characterize the changes in soil moisture and temperature under different land use types. K_a_ was calculated according to the following formula [24]:

$${\text{K}}_{\text{a}}=\frac{x_{\text{max}}}{x_{\text{min}}}$$ (1)

where x_max_ and x_min_ represent the maximum and minimum values of soil moisture (or soil temperature) in the same soil layer, respectively. The higher the K_a_, the greater the variation in soil moisture and temperature, and the lower the K_a_, the lower the variation.

C_v_ is known as a discrete coefficient in statistics and is defined as the ratio of standard deviation (σ) to the mean (μ). According to Abdi [25], C_v_ was calculated as follows:

$${\text{C}}_{\text{v}}=\frac{\sigma }{\mu }$$ (2)

The variability in soil moisture and temperature is reflected in the C_v_. The greater the variation in soil moisture and temperature, the higher the C_v_, and vice versa.

#### Approximate entropy algorithm

The procedure for calculating the approximate entropy (ApEn) [26] is as follows:

Assume that the original data series is *x*(1), *x*(2),…, *x*(N), with a total number of N points.

(1) A set of m-dimensional vectors is composed in sequential order of serial numbers:
$$X\left(\text{i}\right)=[x\left(\text{i}\right),x\left(\text{i}+1\right),\ldots ,x\left(\text{i}+\text{m}-1\right)]\;\;\text{i}=1\sim \text{N}-m+1$$ (3)
(2) The distance d[*X*(i), *X*(j)] between *X*(i) and *X*(j) is defined as the greatest difference between two corresponding elements:
dXi,Xj=xi+k−xj+kk=0~m−1max (4)
  The difference between other corresponding elements in *X*(i) and *X*(j) is less than d. Calculate the distance d[*X*(i), *X*(j)] between *X*(i) and the rest of the vector *X*(j) (j = 1~N−*m*+1, and j≠i) for each i.
(3) Assume a threshold r, count the number of d[*X*(i), *X*(j)] less than r for each i, and calculate the ratio of this number to the total distance N–m, denoted as ${\text{C}}_{\text{i}}^{m}\left(r\right)$, as follows:
$${\text{C}}_{\text{i}}^{m}\left(\text{r}\right)=\frac{1}{\text{N}-m}\left\{\text{Number}\;\text{of}\;\left[\text{d}\left[X\left(\text{i}\right),\;X\left(\text{j}\right)\right]<r\right]\right\}$$ (5)
(4) Calculate the logarithm of ${\text{C}}_{\text{i}}^{m}\left(r\right)$ first, and then calculate the mean value of the logarithm for all i, denoted as Φ^*m*^(*r*), as follows:
$$\emptyset^{m}\left(r\right)=\frac{1}{\text{N}-m+1}\sum_{\text{i}=1}^{\text{N}-m+1}\;\text{ln}\;{\text{C}}_{\text{i}}^{m}\left(r\right)$$ (6)
(5) Add 1 to the dimension to obtain m+1, and repeat steps (1)–(4) to obtain ${\text{C}}_{\text{i}}^{m+1}\left(r\right)$ and Φ^*m*+1^(*r*).
(6) The ApEn of the series can be calculated using the following formula:
$$\text{ApEn}\left(m,r\right)={\text{lim}}_{\text{N}\to \infty }\left[\emptyset^{m}\left(r\right)-\emptyset^{m+1}\left(r\right)\right]$$ (7)
  When N is finite, an estimated ApEn of the series can be obtained as follows:
$$\text{ApEn}\left(m,r,\text{N}\right)=\emptyset^{m}\left(r\right)-\emptyset^{m+1}\left(r\right)$$ (8)
  The parameters *m* and *r* have an impact on estimating ApEn and need to be determined. According to Pincus’s proposal [27], the parameter *m* is set to 2, the parameter *r* is usually *k* times the standard deviation of the data series, and the value of *k* ranges from 0.10 to 0.20, with a step size of 0.01. The influence of different *k* values on the ApEn of the soil moisture and temperature series under the experimental sloping land use types was programmed and calculated using MATLAB R2015b software (The Mathworks, Natick, MA, USA).

## Results

### Descriptive statistics-based variations in soil moisture and temperature

#### Characteristics of soil moisture variation

The changing amplitude and degree of variation of soil moisture under all four land use types in the 2014 and 2015 growing seasons appeared to decrease with a deepening soil layer (Table 2). During the 2014 growing season, the changing amplitude of soil moisture in the surface, middle, and deep layers followed the same trend: K_a (maize)_ <K_a (grassland)_ <K_a (soybean)_ <K_a (jujube)_, and the degree of variation of soil moisture followed the same trend: C_v (maize)_ < C_v (grassland)_ < C_v (soybean)_ < C_v (jujube)_. In the 2015 growing season, the changing amplitude of soil moisture in the surface and middle layers of the four land use types showed the trend K_a (maize)_ <K_a (grassland)_ <K_a (soybean)_ <K_a (jujube)_, and the degree of variation for soil moisture also showed a similar result. The soil moisture in the deep soil layers of the jujube orchard presented a higher changing amplitude and degree of variation than other land use types. Although there was a significant difference in precipitation between the two years, the maize terraced field maintained a low changing amplitude and degree of variation of soil moisture and maintained stable soil moisture conditions, while the jujube orchard showed a greater variation in soil moisture in all soil layers.

**Table 2:** Statistical analysis of variation of soil moisture in profile for the four land use types.

Years	Land use types	Soil layer								
		Surface layer (0–20 cm)			Middle layer (20–60 cm)			Deep layer (60–160 cm)		
		Mean(%)	K_a_	C_v_	Mean(%)	K_a_	C_v_	Mean(%)	K_a_	C_v_
2014	Soybean	14.6	2.33	0.18	12.9	1.69	0.14	13.9	1.16	0.04
	Maize	14.9	1.49	0.09	14.9	1.37	0.07	12.7	1.11	0.03
	Jujube	11.2	2.80	0.27	11.3	2.09	0.16	13.5	1.27	0.07
	Grassland	15.0	2.15	0.18	16.8	1.37	0.08	15.3	1.14	0.04
2015	Soybean	9.9	3.20	0.33	8.8	1.93	0.22	10.6	1.22	0.07
	Maize	12.3	1.74	0.12	12.3	1.49	0.09	12.4	1.26	0.09
	Jujube	8.1	3.60	0.34	7.5	1.94	0.22	12.2	1.63	0.20
	Grassland	10.0	3.08	0.27	11.1	1.85	0.21	12.0	1.48	0.12

The soil moisture active layer was defined as a continuous soil layer with a C_v_ of soil moisture greater than 0.1 in each layer [28]. In the 2014 growing season, the soil moisture active layers of both soybean sloping field and jujube orchard were 0–60 cm in depth. The grassland had a thin soil moisture active layer of 0–20 cm. The C_v_ of the maize terraced field for all layers was less than 0.1, indicating that the soil moisture was relatively stable. During the 2015 growing season, the soil moisture active layer in the jujube orchard and grassland dropped to 160 cm, while that in the maize terraced field was at 0–20 cm.

#### Characteristics of soil temperature variation

The changing amplitude and degree of variation in soil temperature decreased as the soil depth increased (Table 3). For the surface and middle soil layers, variations in the soil temperature of the four land use types were consistent in both growing seasons. The K_a_ and C_v_ of the soil temperature for the jujube orchard were higher than those for the other three land use types and it maintained the minimum temperature of all land use types. The maize terraced field showed the lowest variation in the surface soil layer. Therefore, jujube orchard increased the changing amplitude and degree of variation in soil temperature in the 0–100 cm soil layer during the growing seasons of 2014 and 2015, whereas the maize terraced field decreased the changing amplitude and degree of variation in soil temperature.

**Table 3:** Statistical analysis of variation of soil temperature in profile for the four land use types.

Years	Land use types	Soil layer								
		Surface layer (0–20 cm)			Middle layer (20–60 cm)			Deep layer (60–100 cm)		
		Mean (°C)	K_a_	C_v_	Mean (°C)	K_a_	C_v_	Mean (°C)	K_a_	C_v_
2014	Soybean	14.6	2.59	0.20	12.9	1.88	0.16	13.9	1.86	0.15
	Maize	14.9	2.21	0.20	14.9	1.58	0.11	12.7	1.44	0.09
	Jujube	11.2	2.91	0.23	11.3	1.91	0.17	13.5	1.89	0.16
	Grassland	15.0	2.65	0.20	16.8	1.87	0.16	15.2	1.72	0.14
2015	Soybean	9.9	3.97	0.23	8.8	2.31	0.17	10.6	1.64	0.14
	Maize	12.3	3.57	0.22	12.3	1.86	0.16	12.4	1.84	0.15
	Jujube	8.1	5.85	0.27	7.5	2.34	0.19	12.2	2.12	0.18
	Grassland	10.0	3.91	0.22	11.1	1.88	0.15	12.0	1.77	0.14

### Approximate entropy-based variations in soil moisture and temperature

#### Complexity characteristics of soil moisture changes

**Fig 2:**
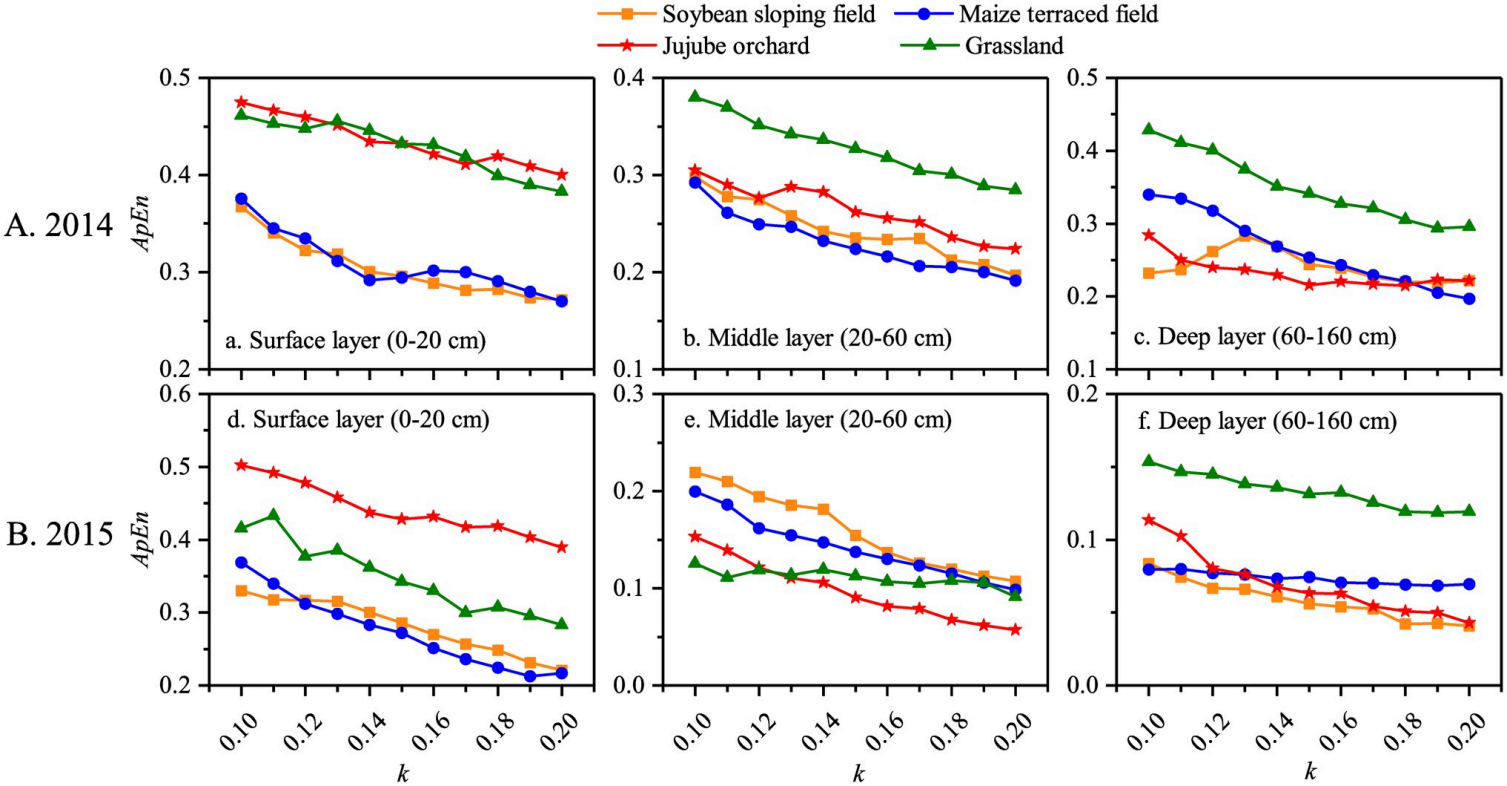
Variation of ApEn of soil moisture series with *k* under the four land use types in the growing seasons of 2014 and 2015.

ApEn was used to measure the complexity of the daily average soil moisture content series in the surface (0–20 cm), middle (20–60 cm), and deep (60–160 cm) layers of soybean sloping field, maize terraced field, jujube orchard, and grassland. As mentioned in Eq 8, the value of ApEn is modulated by the parameter *k*; thus, it need to be determined. The influence of *k* on the ApEn of the soil moisture series under the four land use types is shown in Fig 2. With an increase in *k*, the ApEn of the soil moisture series in the surface, middle, and deep layers under the four land use types showed a decreasing trend in both growing seasons. To determine the parameter *k*, we followed the methodology of Yan and Gao [29], who suggested that the optimal *k* could be selected when changes had minimal perturbation effects on ApEn.

**Fig 3:**
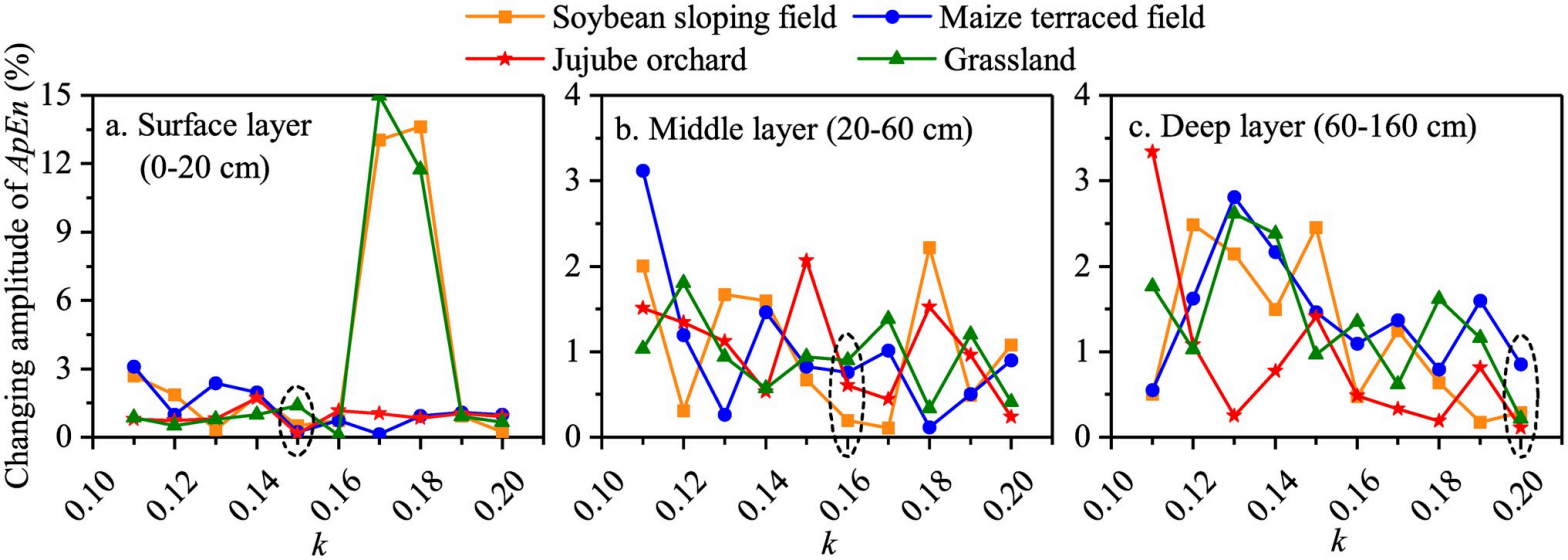
The changing amplitude of ApEn of soil moisture varies with *k* under the four land use types in the 2014 growing season.

In 2014, for the surface layer, a minimum change of only 0.6% was observed for the ApEn of the soil moisture series when *k* was set to 0.15 (Fig 3a). For the middle layer, when *k* was 0.16, the ApEn of the soil moisture of soybean, maize, jujube, and grassland fields changed by 0.19%, 0.75%, 0.60%, and 0.89%, respectively (Fig 3b). Similarly, for the deep layer, the changes in the amplitude of ApEn of soil moisture under the four land use types was minimal when *k* was 0.20 (Fig 3c). As a result, when *k* was set to 0.15, 0.16, and 0.20 for the surface, middle, and deep layers, respectively, the calculation results were the most reliable and robust.

Under the four experimental land use types, ApEn (2, 0.15SD), ApEn (2, 0.16SD), and ApEn (2, 0.20SD) were used to evaluate the complexity of soil moisture in the surface, middle, and deep layers, respectively. During the 2014 growing season, the lowest ApEn of soil moisture in the surface and middle layers was observed in the maize terraced field (Fig 2a and 2b), indicating that the soil moisture during the growing season was stable. Conversely, a high ApEn in grassland in the 20–160 cm soil layer revealed that the soil moisture content was highly variable with precipitation and percolation (Fig 2b and 2c).

**Fig 4:**
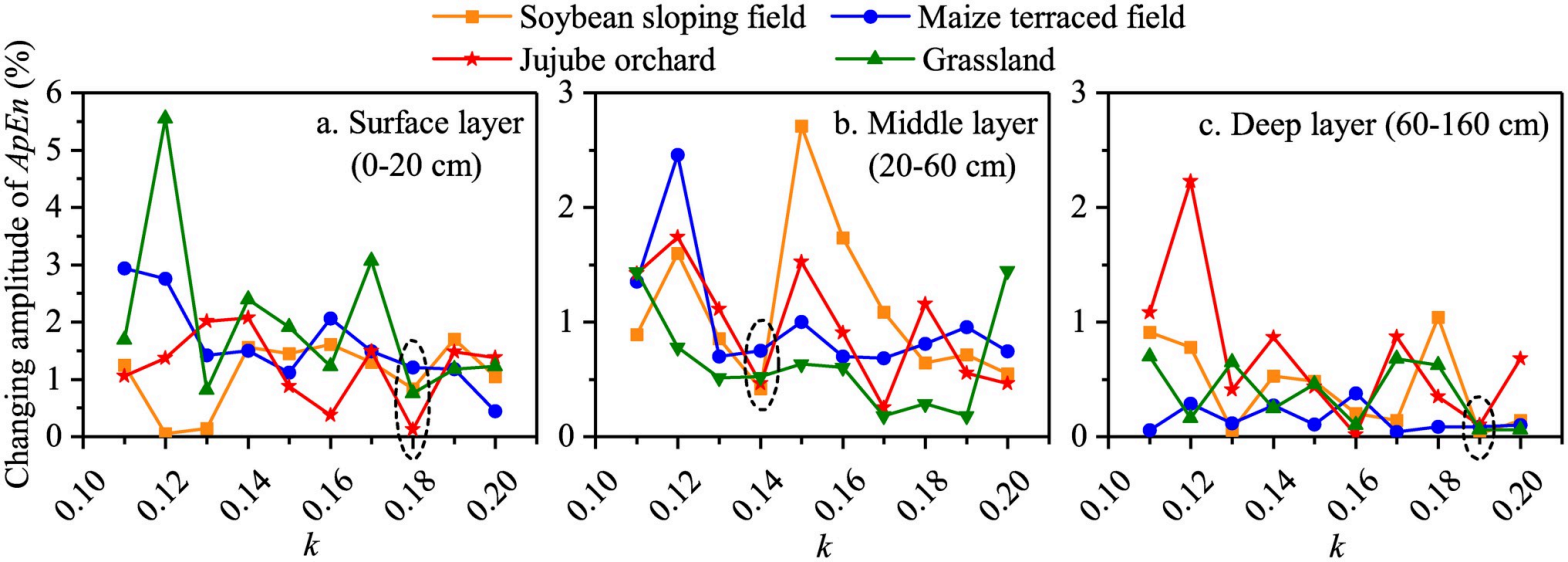
The changing amplitude of ApEn of soil moisture series varies with *k* under the four land use types in the 2015 growing season.

In 2015, for the surface layer, when *k* increased to 0.18, the ApEn of the soil moisture of soybean, maize, jujube, and grassland fields varied by 0.83%, 1.21%, 0.12%, and 0.75%, respectively, with a relatively small average changing amplitude of 0.73% (Fig 4a). The changing amplitude of the ApEn of the soil moisture under the four land use types was in the range of 0.42%–0.75% in the middle layer, when *k* was 0.14 (Fig 4b). For the deep layer, when *k* was 0.19, the ApEn of the soil moisture of soybean, maize, jujube, and grassland fields changed by 0.02%, 0.08%, 0.10%, and 0.06%, respectively, and the average changing amplitude was only 0.07%, which was lowest achieved in this study (Fig 4c). As such, the results were relatively reliable when the *k* values for the surface, middle, and deep layers were 0.18, 0.14, and 0.19, respectively. ApEn (2, 0.18SD), ApEn (2, 0.14SD), and ApEn (2, 0.19SD) were thus used to evaluate soil moisture complexity in the surface, middle, and deep layers under the four land use types, respectively.

The results of the ApEn of soil moisture in the surface layer during the 2015 growing season were consistent with those in 2014, with the highest and lowest ApEn of soil moisture being observed in jujube orchard and maize terraced field, respectively (Fig 2a and 2d). The ApEn of the soybean field was the highest in the middle layer at approximately 0.18, whereas the ApEn of the other three land use types was all lower than 0.15 (Fig 2e). No significant differences were observed in the deep layer for the soybean, maize, and jujube fields (Fig 2f).

#### Complexity characteristics of soil temperature change

**Fig 5:**
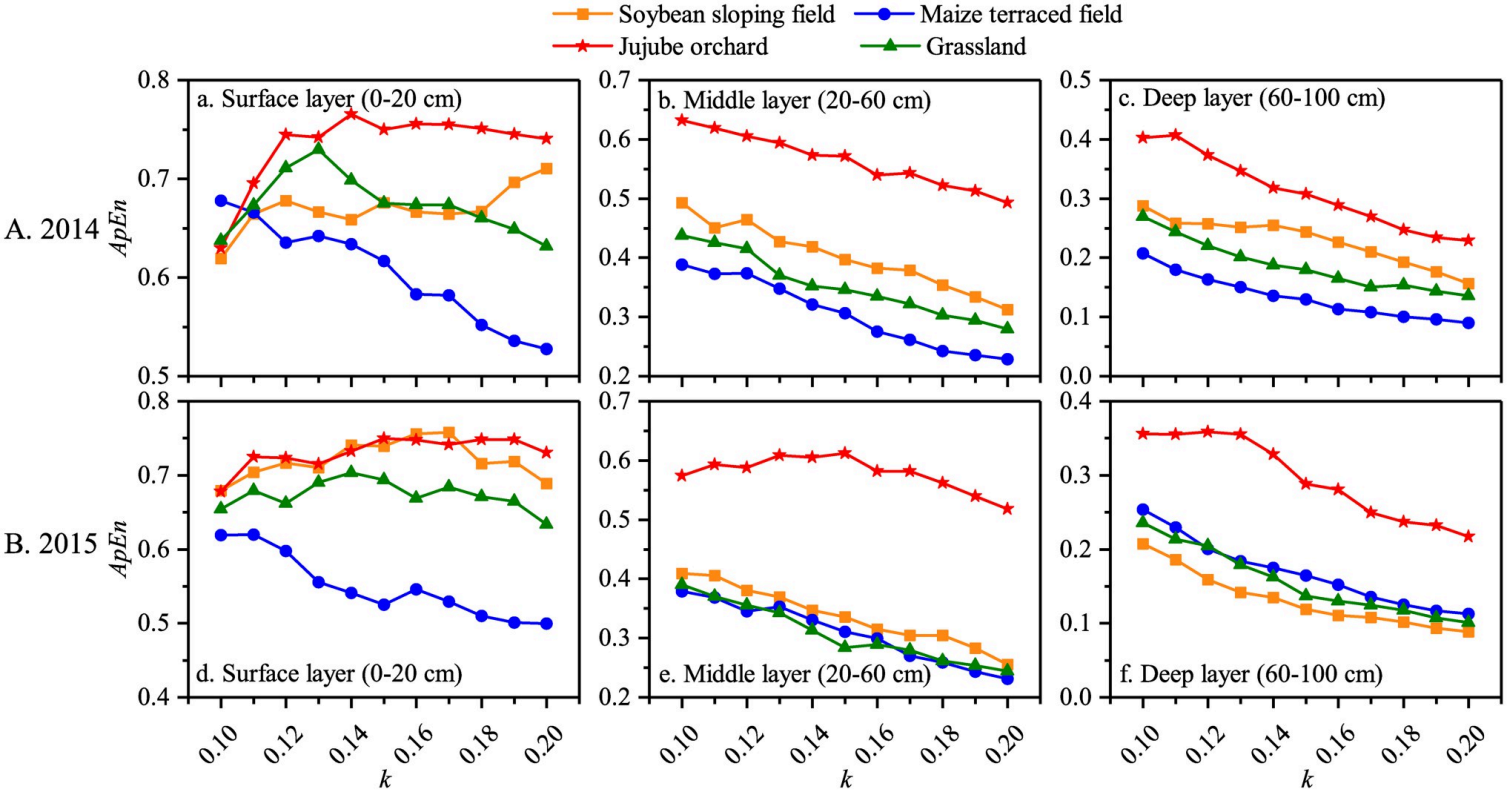
Variation of ApEn of the soil temperature with *k* under the four land use types in the growing seasons of 2014 and 2015.

The complexity of the daily average soil temperature in the surface layer (0–20 cm), middle layer (20–60 cm), and deep layer (60–100 cm) under the four land use types was also estimated using approximate entropy. The ApEn of soil temperature in the surface layer of the grassland first increased and then decreased with an increase in *k*, whereas the ApEn of the soybean field and jujube orchard showed a gradually increasing trend (Fig 5a and 5d). With the increase in *k*, the ApEn of soil temperature in the surface layer of the maize terraced field, as well as that in the middle and deep layers under the four land use types, all showed a decreasing trend (Fig 5).

**Fig 6:**
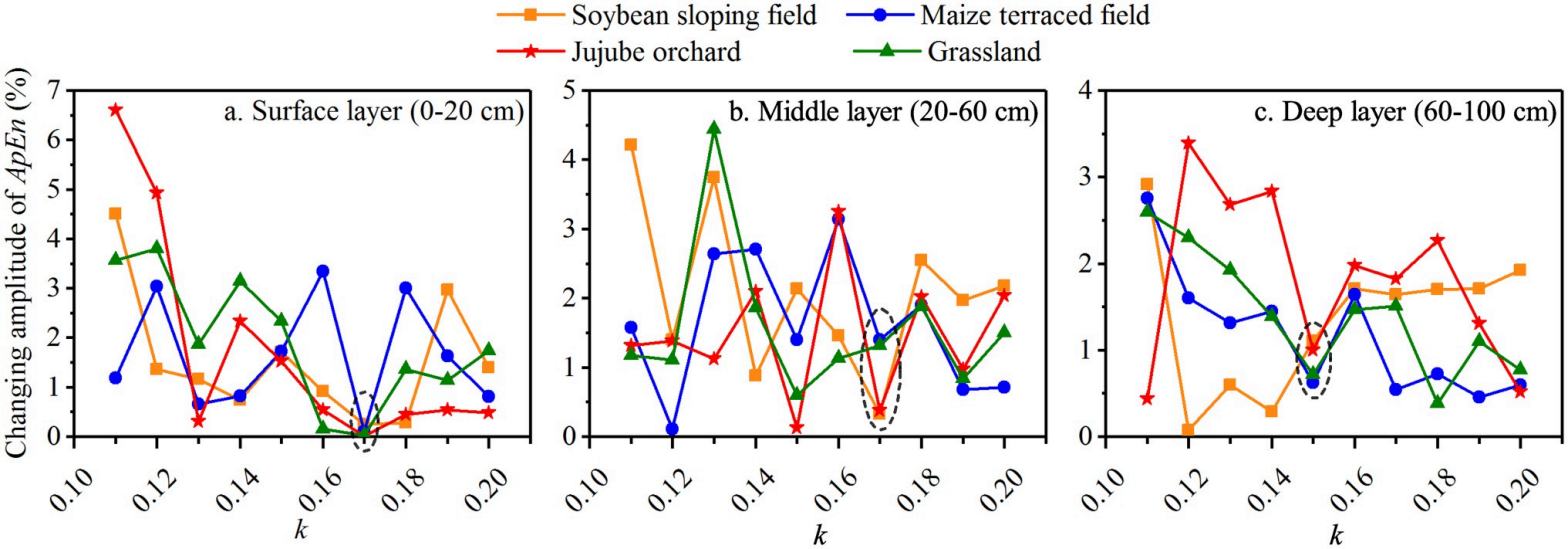
The changing amplitude of ApEn of soil temperature series varies with *k* under the four experimental sloping land use types in the 2014 growing season.

The changing amplitude of the ApEn of soil temperature with *k* under different land use types was calculated, and the optimal *k* was determined by the minimum changing amplitude. The results showed that the average changing amplitude of the ApEn of soil temperature in the surface and middle layers under the four land use types in the 2014 growing season were 0.10% and 0.85%, respectively, when *k* was 0.17 (Fig 6a and 6b). When *k* was 0.15, the changing amplitude of the ApEn of soil temperature in the deep layer under soybean, maize, jujube, and grassland fields was 1.11%, 0.63%, 1.00%, and 0.72%, respectively, indicating that the calculation results were reliable (Fig 6c). Finally, the values of parameter *k* were set to 0.17, 0.17, and 0.15 for the surface, middle, and deep layers, respectively.

The complexity of the soil temperature series in the surface layer under the four land use types during the 2014 growing season followed the trend: ApEn _(jujube)_ > ApEn _(grassland)_ > ApEn _(soybean)_ > ApEn _(maize)_ (Fig 5a). The complexity in the middle layer presented the same results, with the highest being in the jujube orchard and the lowest being in the maize field (Fig 5b). For each land use type, ApEn (2, 0.15SD) was used to evaluate the complexity of soil temperature in the deep layer, and the complexity ranking of the deep layer soil temperature was consistent with that in the middle layer (Fig 5b and 5c). It can be concluded that in the 2014 growing season, the complexity of soil temperature in the 0–100 cm soil layer of the jujube orchard was the greatest, and that in the maize terraced field was the lowest.

**Fig 7:**
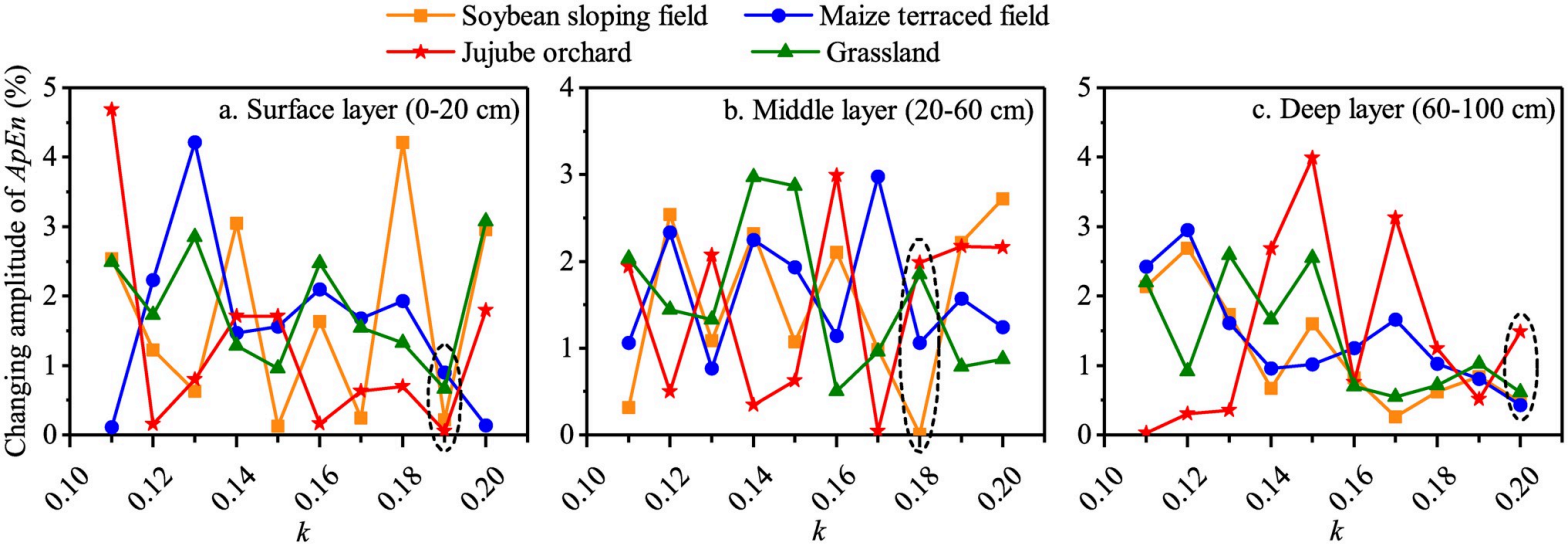
The changing amplitude of ApEn of soil temperature series varies with *k* under the four experimental sloping land use types in the 2015 growing season.

During the 2015 growing season, when *k* was 0.19, 0.18, and 0.20, the changing amplitude of the ApEn of soil temperature in the surface, middle, and deep layers, respectively, under the four land use types was the lowest, and the calculation result was the most reliable (Fig 7). As a result, the complexity of soil temperature in the surface, middle, and deep layers under each sloping land use type was calculated using ApEn (2, 0.19SD), ApEn (2, 0.18SD), and ApEn (2, 0.20SD). Under the four land use types in the 2015 growing season, the complexity of soil temperatures in the surface and middle layers was as follows: ApEn _(jujube)_ > ApEn _(soybean)_ > ApEn _(grassland)_ > ApEn _(maize)_ (Fig 5d and 5e). The ApEn of the deep layer soil temperature series in the soybean sloping field, maize terraced field, and grassland were all approximately 0.1, lower than the ApEn of the jujube orchard (approximately 0.22) (Fig 5f). The soil temperature in the jujube orchard in the 0–100 cm soil layer was the most complex in the 2015 growing season, whereas it was quite stable in the maize terraced field.

## Discussion

### Characteristics of variation in soil moisture and temperature

Soil moisture in the loess hilly region is variable because of the combined effects of precipitation, evaporation, transpiration, runoff, and other factors [30–33]. The complex underlying surface of the loess hilly region and various land use types vary, resulting in complex spatial changes in soil moisture evapotranspiration [8]. Soil temperature is controlled by soil properties, meteorological conditions (precipitation, evapotranspiration, air temperature, solar radiation, etc.), topography (slope aspect, slope gradient, slope position, etc.), and vegetation (coverage, species, etc.), with complex variation characteristics [34–36]. In arid and semi-arid regions, land use is a critical factor that influences soil temperature changes [7, 37].

The changing amplitude, degree of variation, and active layer of soil moisture were all the lowest in the maize terraced field, according to this study (Table 2). The terraced fields incorporate a portion of the slope runoff into the soil during precipitation period, converting non-ponding infiltration into ponding infiltration, or non-pressurized infiltration into pressurized infiltration, thus reducing soil moisture volatility [38]. During the growing season, the maize terraced field decreased the changing amplitude and degree of variation of soil temperature (Table 3), which is not only beneficial for the growth of vegetation roots but also plays an important role in the activity of soil microorganisms and the conversion of materials and energy [39, 40]. The maize terraced fields had higher soil moisture levels during the growing season, and water has a much higher specific heat capacity (4.2×10^3^ J/(kg∙°C)) than soil (1.0×10^3^−2.5×10^3^ J/(kg∙°C)). This means that the heat absorbed by the soil is much less than that absorbed by water when the temperature rises by the same amount. However, during the cooling process, the soil loses heat more easily than water. As a result, for certain soil types, the soil specific heat capacity increases as the soil moisture content increases. The higher the soil moisture content, the slower the temperature rise and fall [41]. Thus, terraced fields can control and maintain soil temperatures. The changing amplitude and degree of variation of soil moisture and temperature in the jujube orchard were relatively high in the growing seasons of 2014 and 2015 (Tables 2 and 3). This phenomenon was caused by the wide row spacing of jujube trees, which resulted in low vegetation coverage and large areas of bare land. As precipitation and evapotranspiration are the primary factors that influence soil moisture, local external factors such as weather can easily influence the soil moisture content. Frequent drying and wetting can cause significant changes in soil moisture. Furthermore, the soil surface of the jujube orchard was bare, and the soil temperature was significantly affected by solar radiation, fluctuating dramatically. In addition, bare soil promotes evaporation and has a low moisture content, resulting in a low heat capacity and a weak soil temperature regulation effect.

### Application of approximate entropy in the study of the complex characteristics of soil moisture and temperature

Traditional statistical methods (such as K_a_ and C_v_) are typically used to evaluate soil moisture and temperature variations, and the calculation process is simple [23, 42]. However, the sample size, or the length of the data series, has a significant impact on the reliability of K_a_ and C_v_. The accuracy of the K_a_ and C_v_ evaluation results improves as the sample size increases [13]. Datasets obtained in field experiments, on the other hand, are limited. The biggest advantage of approximate entropy as a tool for quantifying the complexity of a time series is that the calculation result from a small amount of data is comparable to that from a large amount of data; there is no requirement for substantial amounts of data, allowing robust estimates from a short data series [43]. Furthermore, approximate entropy can remove some interference signals, has strong anti-noise and anti-interference capabilities, and has a broad range of applications with few limitations [44, 45]. During the 2014 and 2015 growing seasons, approximate entropy was used to assess the complexity of soil moisture and temperature under four different land use types in the loess hilly region. The approximate entropy values of soil moisture and temperature at different depths under the four sloping land use types had the following order: ApEn _(surface layer)_> ApEn _(middle layer)_> ApEn _(deep layer)_ (Figs 2 and 5). This indicates that the impact of external environmental conditions on soil moisture and temperature is more pronounced closer to the soil surface, and changes in soil moisture and temperature are more complicated. As the soil depth increases, the impact of environmental factors on soil moisture and temperature decreases, as does the complexity of soil moisture and temperature changes. This finding is in line with that of Wang, who used approximate entropy to investigate the complex characteristics of soil moisture content and soil temperature at different depths in a snow-soil composite system, and found that the soil moisture content and temperature varied the most in the surface layer with complex variations [19]. The findings of this study are consistent the current situation. The surface soil is in close contact with the atmosphere and is easily affected by precipitation replenishment and evaporation, as well as by a large amount of solar radiation, which causes heat exchange and transfer to be violent, resulting in a high degree of soil moisture and temperature changes [46–49].

Because the groundwater in the loess hilly region lies deep underground and the deep soil can only obtain moisture and heat from the surface soil, the variations in deep soil are generally low [50]. The approximate entropy of the jujube orchard was relatively high during the two growing seasons (Figs 2 and 5), indicating that factors affected the soil moisture and temperature the most in the jujube orchard, with many uncertain components, resulting in the high structural complexity of the soil hydrothermal system. The approximate entropy of the maize terraced field was relatively low, regardless of the amount of precipitation received during the growing season, indicating that relatively few factors influenced the soil moisture and temperature series in the maize terraced field, and that uncertainty was relatively low, reducing the structural complexity of the related soil hydrothermal system. Finally, the approximate entropy-based complexity of soil moisture and temperature agrees with the descriptive statistical analysis, demonstrating that the approximate entropy algorithm is stable and reliable, and that it can theoretically verify the complexity of soil moisture and temperature changes under different sloping land use types. This further indicates that sloping land use types have a significant impact on soil moisture and temperature changes in the loess hilly region.

### Suggestions for optimizing sloping land use

In the loess hilly region, severe soil erosion is the main bottleneck impeding ecological construction and sustainable economic and social development, with vegetation restoration and reconstruction being viable methods to prevent soil erosion [51, 52]. Appropriate soil moisture and temperature conditions are beneficial for vegetation growth and restoration [53, 54]. The soil moisture and temperature fluctuated violently in the jujube orchard in this study (Tables 2 and 3), and the variations were complex, which is not conducive to jujube root growth and is likely to inhibit jujube tree growth and development. Thus, it is critical to take appropriate soil management measures to adjust the jujube orchard’s soil moisture and temperature, and to reduce the risk of jujube tree growth caused by drastic changes in soil moisture and temperature. Mulching has obvious effects on soil moisture conservation and temperature regulation as a dry farming technique [55]. Straw mulching and jujube branch mulching, according to existing research on jujube orchard mulching measures, induce a high water-holding capacity and a strong temperature-regulating effect, ensuring an appropriate moisture and temperature environment for jujube roots [56, 57]. Crop straw and pruned jujube branches can be used for this purpose, which saves money on mulching and reduces pollution caused by the burning of straw. This has some application value for the synchronized improvement of economic benefits and ecological functions for dry-farming jujube orchards in the loess hilly region. Furthermore, pruning can be conducted based on the physiological characteristics of jujube trees, as well as local ecological and climatic conditions, to maintain tree specifications within a reasonable range, which can reduce not only the interception of precipitation by the canopy but also the transpiration, which is beneficial for preserving soil moisture [58]. Moreover, water and soil conservation measures can be implemented in jujube orchards, such as level terraced fields and fish-scale pits, to collect a portion of the runoff during precipitation and increase the soil moisture content [59]. All of the aforementioned methods can be used to regulate soil temperature using water.

According to this study, the maize terraced field suppressed soil temperature fluctuations and balanced the dramatic changes in soil moisture caused by uneven precipitation during the growing season (Tables 2 and 3). Owing to their topographical advantages, terraced fields have a high water-storage capacity, resulting in a high soil moisture content, leading to a high heat capacity, thermal conductivity, and latent heat of evaporation, additionally affecting soil reflectivity [60–62]. A suitable soil moisture content in terraced fields is conducive to the absorption of heat in the daytime and the decrease in heat loss at night, thereby making the soil’s hydrothermal environment generally stable. Considering the common goal of sustainable sloping field utilization and high-efficiency natural precipitation utilization in the loess hilly region, the transformation of sloping fields into terraced fields is advantageous for retaining natural precipitation, reducing surface runoff, expanding rainwater infiltration, improving precipitation utilization, and regulating soil temperature through soil moisture. This is critical for guiding sloping field management and development, as well as for ecological agriculture.

### Deficiencies and prospects

Underlying surface conditions were strictly controlled in this study because we focused on the effects of vegetation and micro-topography on soil moisture and temperature. The selected soybean sloping field, maize terraced field, jujube orchard, and grassland had similar altitudes, slope aspects, and gradients. However, under actual field conditions, even in a small area, two sloping fields may have differences in micro-topography, vegetation, and gradient. As a result, the study’s experimental conditions were ideal. In the future, the impact of various underlying surface conditions on soil moisture and temperature changes, such as new and promising sloping land use types (jujube-crop intercropping, apple-crop compounding, etc.), will need to be considered.

Because soil moisture and temperature conditions are heavily influenced by meteorological conditions (precipitation, air temperature, etc.), the long-term continuous monitoring of soil moisture and temperature is recommended. However, because the soybean sloping field was levelled and original maize in the terraced field was replaced with sweet potatoes in May 2016, the original experimental scheme had to be terminated due to significant changes in the research subjects. Therefore, this study only examines the normal precipitation and dry years, and there is no monitoring data for the precipitation in other years.

More theories and methods for characterizing complexity, such as information coefficients, symbolic dynamics, chaos theory, and complex networks, should also be used to assess the complexity of soil moisture and temperature changes. The feasibility of different methods for typical sloping land use types in the loess hilly region can be determined by analyzing the differences in the evaluation of the complexity of soil moisture and temperature changes using these methods.

## Conclusions

Jujube orchard showed an increase in the changing amplitude, degree of variation, and active layer of soil moisture at 0–160 cm, as well as in the changing amplitude and degree of variation of soil temperature in the 0–100 cm soil layer during the growing seasons of the normal precipitation year (2014) and dry year (2015). The changing amplitude, degree of variation, and active layer of soil moisture all decreased in the maize terraced field, as did the changing amplitude and degree of variation in soil temperature. The approximate entropy of the soil moisture in the 0–20 cm soil layer under the four land use types under a normal precipitation year and dry year followed the order: ApEn _(jujube orchard)_ > ApEn _(grassland)_ > ApEn _(soybean sloping field)_ > ApEn _(maize terraced field)_. The ApEn of soil moisture in the 0–160 cm soil layer of the maize terraced field was the lowest during the 2014 growing season. The ApEn of the soil temperature series in the 0–100 cm layer was the highest for the jujube orchard in the two growing seasons, while it was the lowest in the maize terraced field. In conclusion, the soil moisture and temperature change processes in the jujube orchard were complicated, whereas the changes in the maize terraced field were simple, which was conducive to maintaining soil moisture stability and regulating soil temperature. It is possible to assess the complexity of soil moisture and temperature under typical sloping land use types in the loess hilly region using approximate entropy. The findings reveal the characteristics of soil moisture and temperature under typical sloping land use types in the loess hilly region, which can serve as a theoretical foundation and practical guidance for the efficient and sustainable utilization of sloping land resources in this region.
